# Report of SARS-CoV-2 BA.1 Lineage in Morocco

**DOI:** 10.1128/mra.00169-22

**Published:** 2022-04-13

**Authors:** Safae El Mazouri, Houda Bendani, Nasma Boumajdi, Mhammed Chaoui Roqai, Tarik Aanniz, Myriam Seffar, Hakima Kabbaj, Ghizlane El Amin, Amal Zouaki, Saaïd Amzazi, Mouna Ouadghiri, Lahcen Belyamani, Azeddine Ibrahimi

**Affiliations:** a Medical Biotechnology Laboratory (MedBiotech), Bioinova Research Center, Rabat Medical and Pharmacy School, Mohammed V University, Rabat, Morocco; b Ecole des Hautes Etudes de Biotechnologie et de Santé (EHEB), Casablanca, Morocco; c Laboratoire Central de Virologie, Centre Hospitalo-Universitaire Ibn Sina, Hôpital des Spécialités, Rabat, Morocco; d Laboratory of Human Pathologies Biology, Faculty of Sciences, Mohammed V University, Rabat, Morocco; e Emergency Department, Military Hospital Mohammed V, Rabat, Morocco; f Rabat Medical and Pharmacy School, Mohammed V University, Rabat, Morocco; DOE Joint Genome Institute

## Abstract

Here, we report the near-complete genome sequence and genetic variations of a clinical sample of SARS-CoV-2 for the newly emerged Omicron variant (BA.1). The sample was collected from a nasopharyngeal swab of a Moroccan patient, and the sequencing was done using Ion S5 technology.

## ANNOUNCEMENT

On 26 November 2021, the World Health Organization (WHO) announced the emergence of a new variant of concern (VOC) named Omicron (B.1.1.529), belonging to the *Betacoronavirus* genus of the *Coronaviridae* family. Currently, Omicron harbors 32 mutations in the spike protein (S) (1, 2). Moreover, the Omicron variant shows more vaccine evasion and a higher rate of reinfection compared to previous VOCs (3). Monitoring the genomic diversity of SARS-CoV-2 remains pivotal in detecting mutations, their distribution, and their potential impact (4). In this study, near-complete genome sequencing of a SARS-CoV-2 strain was carried out using Ion S5 sequencing technology (5).

The sampling was carried out on 27 December 2021. RNA was extracted from a nasopharyngeal swab sample from a patient in the Rabat-Salé-Kenitra Region, Morocco, at the Hospital Ibn Sina of Rabat, using the MegaPure virus DNA/RNA purification kit in the nucleic acid purification system-32 (Bigfish Bio-tech, Hangzhou, China). The patient was identified as positive for COVID-19 by reverse transcriptase quantitative PCR using a SARS-CoV-2 kit (MAScIR, Morocco) and exhibited cycle threshold (*C_T_*) values of 18 and 19 for the RdRp and S genes, respectively. Total cDNA was prepared using a SuperScript VILO cDNA synthesis kit (Invitrogen, Thermo Fisher Scientific, USA) and used for SARS-CoV-2 library preparation with an Ion AmpliSeq kit for Chef DL8 (Thermo Fisher Scientific). The library was adjusted to 30 picomoles (pM) and loaded onto the Ion Chef instrument (Thermo Fisher Scientific) for emulsion PCR, enrichment, and loading onto the Ion S5 530 chip. Whole-genome sequencing (WGS) was performed using the Ion AmpliSeq SARS-CoV-2 research panel (Invitrogen, Thermo Fisher Scientific) for complete viral genome sequencing according to the instructions for use on an Ion GeneStudio S5 Prime series system.

Raw data consisting of 981,092 reads with an average read length of 200 bp were analyzed using Torrent Suite v5.12.0 software. The NGS QC Toolkit v2.3.3 was used to remove low-quality and short reads. The consensus sequence was generated using IRMAreport v1.3.0.2 and mapped to the reference sequence (GenBank accession number MN908947.3) using Minimap (6). The BAM file was sorted using SAMtools sort (7); then, it was used to call the genetic variations in variant call format with SAMtools mpileup and bcftools using the multiallelic-caller option. We then annotated the file, and its impact was predicted using SnpEff (8). All tools were run with default parameters.

Our analysis allowed us to obtain a near-complete SARS-CoV-2 genome with a length of 29,805 bp and an overall DNA G+C content of 37.96%. A total of 974,515 reads were correctly mapped, covering 99.33% of the total genome with a mean depth of 1,995×.

Phylogenetic analysis using Phylogenetic Assignment of Named Global Outbreak Lineages (pangolin) (9) revealed that the strain belongs to lineage BA.1. The genetic variation process revealed a total of 61 variations compared to the reference sequence (GenBank accession number MN908947.3) (Table 1).

**TABLE 1:** Types and effects of identified gene variations

Gene	Nucleotide position	Nucleotide change	Residue change	Effect
ORF1ab	241	-25C>T	No change assigned	upstream_gene_variant
	2015	1750A>G	Met584Val	missense_variant
	2229	1964G>A	Cys655Tyr	missense_variant
	2832	2567A>G	Lys856Arg	missense_variant
	3037	2772C>T	Phe924Phe	synonymous_variant
	5386	5121T>G	Ala1707Ala	synonymous_variant
	6512	6248_6250delGTT	Ser2083_Leu2084delinsIle	disruptive_inframe_deletion
	8393	8128G>A	Ala2710Thr	missense_variant
	10029	9764C>T	Thr3255Ile	missense_variant
	10449	10184C>A	Pro3395His	missense_variant
	11282	11022_11030delGTCTGGTTT	Leu3674_Gly3676del	disruptive_inframe_deletion
	11537	11272A>G	Ile3758Val	missense_variant
	13195	12930T>C	Val4310Val	synonymous_variant
	14408	14144C>T	Pro4715Leu	missense_variant
	15240	14976C>T	Asn4992Asn	synonymous_variant
	18163	17899A>G	Ile5967Val	missense_variant
S	21762	200C>T	Ala67Val	missense_variant
	21764	204_209delACATGT	His69_Val70del	disruptive_inframe_deletion
	21846	284C>T	Thr95Ile	missense_variant
	21986	425_433delGTGTTTATT	Gly142_Tyr145delinsAsp	disruptive_inframe_deletion
	22578	1016G>A	Gly339Asp	missense_variant
	22673	1111T>C	Ser371Pro	missense_variant
	22674	1112C>T	Ser371Phe	missense_variant
	22679	1117T>C	Ser373Pro	missense_variant
	22686	1124C>T	Ser375Phe	missense_variant
	22813	1251G>T	Lys417Asn	missense_variant
	22882	1320T>G	Asn440Lys	missense_variant
	22898	1336G>A	Gly446Ser	missense_variant
	22992	1430G>A	Ser477Asn	missense_variant
	22995	1433C>A	Thr478Lys	missense_variant
	23013	1451A>C	Glu484Ala	missense_variant
	23040	1478A>G	Gln493Arg	missense_variant
	23048	1486G>A	Gly496Ser	missense_variant
	23055	1493A>G	Gln498Arg	missense_variant
	23063	1501A>T	Asn501Tyr	missense_variant
	23075	1513T>C	Tyr505His	missense_variant
	23202	1640C>A	Thr547Lys	missense_variant
	23403	1841A>G	Asp614Gly	missense_variant
	23525	1963C>T	His655Tyr	missense_variant
	23599	2037T>G	Asn679Lys	missense_variant
	23604	2042C>A	Pro681His	missense_variant
	23854	2292C>A	Asn764Lys	missense_variant
	23948	2386G>T	Asp796Tyr	missense_variant
	24130	2568C>A	Asn856Lys	missense_variant
	24424	2862A>T	Gln954His	missense_variant
	24469	2907T>A	Asn969Lys	missense_variant
	24503	2941C>T	Leu981Phe	missense_variant
	25000	3438C>T	Asp1146Asp	synonymous_variant
ORF3a	25584	192C>T	Thr64Thr	synonymous_variant
E	26270	26C>T	Thr9Ile	missense_variant
M	26530	8A>G	Asp3Gly	missense_variant
	26577	55C>G	Gln19Glu	missense_variant
	26709	187G>A	Ala63Thr	missense_variant
ORF6	27259	58A>C	Arg20Arg	synonymous_variant
ORF8	27807	27807C>T	No change assigned	intergenic_region
N	28271	28271A>T	No change assigned	intergenic_region
	28311	38C>T	Pro13Leu	missense_variant
	28361	90_98delAGAACGCAG	Glu31_Ser33del	disruptive_inframe_deletion
	28881	608G>A	Arg203Lys	missense_variant
	28882	609G>A	Arg203Arg	synonymous_variant
	28883	610G>C	Gly204Arg	missense_variant

### Data availability.

This sequence was deposited at GenBank under the accession number OM432158.1. The raw reads were deposited at the NCBI Sequence Read Archive (SRA) under the accession number SRR17818130.

## References

[1] Gao SJ, Guo H, Luo G. 2022. Omicron variant (B. 1.1. 529) of SARS‐CoV‐2, a global urgent public health alert! J Med Virol 94:1255–1256. doi:10.1002/jmv.27491. 34850421 PMC9015397

[2] Pascarella S, Ciccozzi M, Bianchi M, Benvenuto D, Cauda R, Cassone A. 2022. The electrostatic potential of the Omicron variant spike is higher than in Delta and Delta‐plus variants: a hint to higher transmissibility? J Med Virol 94:1277–1280. doi:10.1002/jmv.27528. 34914120

[3] Pulliam JR, van Schalkwyk C, Govender N, von Gottberg A, Cohen C, Groome MJ, Dushoff J, Mlisana K, Moultrie H. 2021. Increased risk of SARS-CoV-2 reinfection associated with emergence of the Omicron variant in South Africa. medRxiv doi:10.1101/2021.11.11.21266068. PMC8995029 35289632

[4] Akkiz H. 2021. Implications of the novel mutations in the SARS-CoV-2 genome for transmission, disease severity, and the vaccine development. Front Med (Lausanne) 8:636532. doi:10.3389/fmed.2021.636532. 34026780 PMC8137987

[5] Lopez-Rincon A, Perez-Romero CA, Tonda A, Mendoza-Maldonado L, Claassen E, Garssen J, Kraneveld AD. 2021. Design of specific primer sets for the detection of B.1.1.7, B.1.351 and P.1 SARS-CoV-2 variants using deep learning. bioRxiv doi:10.1101/2021.01.20.427043. PMC7806918 33441822

[6] Li H. 2018. Minimap2: pairwise alignment for nucleotide sequences. Bioinformatics 34:3094–3100. doi:10.1093/bioinformatics/bty191. 29750242 PMC6137996

[7] Li H, Handsaker B, Wysoker A, Fennell T, Ruan J, Homer N, Marth G, Abecasis G, Durbin R, 1000 Genome Project Data Processing Subgroup. 2009. The Sequence Alignment/Map format and SAMtools. Bioinformatics 25:2078–2079. doi:10.1093/bioinformatics/btp352. 19505943 PMC2723002

[8] Cingolani P, Platts A, Wang LL, Coon M, Nguyen T, Wang L, Land SJ, Lu X, Ruden DM. 2012. A program for annotating and predicting the effects of single nucleotide polymorphisms, SnpEff: SNPs in the genome of Drosophila melanogaster strain w1118; iso-2; iso-3. Fly (Austin) 6:80–92. doi:10.4161/fly.19695. 22728672 PMC3679285

[9] O'Toole Á, Scher E, Underwood A, Jackson B, Hill V, McCrone JT, Colquhoun R, Ruis C, Abu-Dahab K, Taylor B, Yeats C, Du Plessis L, Maloney D, Medd N, Attwood SW, Aanensen DM, Holmes EC, Pybus OG, Rambaut A. 2021. Assignment of epidemiological lineages in an emerging pandemic using the pangolin tool. Virus Evol 7:veab064. doi:10.1093/ve/veab064. 34527285 PMC8344591

